# A New Yeast Poly(A) Polymerase Complex Involved in RNA Quality Control

**DOI:** 10.1371/journal.pbio.0030189

**Published:** 2005-04-19

**Authors:** Sˇteˇpánka Vanˇácˇová, Jeannette Wolf, Georges Martin, Diana Blank, Sabine Dettwiler, Arno Friedlein, Hanno Langen, Gérard Keith, Walter Keller

**Affiliations:** **1**Department of Cell Biology, BiozentrumUniversity of Basel, BaselSwitzerland; **2**Roche Genetics, F. Hoffmann-La Roche AGBaselSwitzerland; **3**Institut de Biologie Moléculaire et Cellulaire du CNRS, Université Louis PasteurStrasbourgFrance; University of Massachusetts Medical SchoolUnited States of America

## Abstract

Eukaryotic cells contain several unconventional poly(A) polymerases in addition to the canonical enzymes responsible for the synthesis of poly(A) tails of nuclear messenger RNA precursors. The yeast protein Trf4p has been implicated in a quality control pathway that leads to the polyadenylation and subsequent exosome-mediated degradation of hypomethylated initiator tRNA^Met^ (tRNA_i_^Met^). Here we show that Trf4p is the catalytic subunit of a new poly(A) polymerase complex that contains Air1p or Air2p as potential RNA-binding subunits, as well as the putative RNA helicase Mtr4p. Comparison of native tRNA_i_^Met^ with its in vitro transcribed unmodified counterpart revealed that the unmodified RNA was preferentially polyadenylated by affinity-purified Trf4 complex from yeast, as well as by complexes reconstituted from recombinant components. These results and additional experiments with other tRNA substrates suggested that the Trf4 complex can discriminate between native tRNAs and molecules that are incorrectly folded. Moreover, the polyadenylation activity of the Trf4 complex stimulated the degradation of unmodified tRNA_i_^Met^ by nuclear exosome fractions in vitro. Degradation was most efficient when coupled to the polyadenylation activity of the Trf4 complex, indicating that the poly(A) tails serve as signals for the recruitment of the exosome. This polyadenylation-mediated RNA surveillance resembles the role of polyadenylation in bacterial RNA turnover.

## Abstract 2

A new molecular surveillance mechanism is uncovered in eukaryotes, in which incorrectly folded tRNAs are polyadenylated and then targeted for degradation

## Introduction

The addition of a tract of poly(A) to the 3′ ends of most eukaryotic messenger RNA precursors (pre-mRNAs) is an important step in their processing. Poly(A) tails have multiple functions: they facilitate the export of mRNAs from the nucleus, increase the efficiency of translation initiation, and stabilize the mRNAs in the cytoplasm. 3′ End processing is carried out by a multiprotein machinery and is coupled to transcription by RNA polymerase II and to the other RNA-processing reactions [1–6]. Polyadenylation is catalyzed by poly(A) polymerases (PAPs) that belong to a family of related nucleotidyltransferases [7–9]. Although they can be isolated as single enzymatically active polypeptides, the conventional nuclear PAPs acquire their substrate specificity by association with additional proteins [2,10]. All PAPs require an RNA primer with a free 3′ hydroxyl end to which adenosine monophosphate residues are added from adenosine triphosphate (ATP). The canonical PAPs are U-shaped molecules with a tripartite domain organization [11–13]. The N-terminal domain carries the active site with three highly conserved aspartate residues [9]. The central domain orients the incoming ATP and possibly interacts with the 3′ end of the RNA primer [12]. The C-terminal domain contains an RNA-binding domain, nuclear localization signals, and multiple phosphorylation sites involved in regulating enzyme activity.

In 2002, three groups reported the discovery of a new class of poly(A) polymerases. The new polymerases were Cid1p and Cid13p in the fission yeast Schizosaccharomyces pombe [14,15] and GLD-2 and GLD-3 in Caenorhabditis elegans [16]. Recombinant Cid1p and Cid13p from *S. pombe,* members of the Trf4/5 family of proteins first described in budding yeast [17], were shown to have RNA-dependent PAP activity in vitro [14,15]. The proteins are located in the cytoplasm and have been proposed to stabilize and activate specific mRNAs by extending their poly(A) tails [15]. The same studies also reported that HA-tagged Trf4p from S. cerevisiae had PAP activity in vitro; however, the activity was not characterized in any detail. Earlier reports had indicated that recombinant Trf4p has DNA-dependent DNA polymerase activity [18,19].

The GLD-2 and GLD-3 proteins control different steps of germline development in *C. elegans,* including the mitosis/meiosis decision [20]. The GLD-2 protein contains a domain shared by Trf4p and Trf5p that resembles the catalytic core of nuclear PAPs. GLD-3 belongs to the bicaudal-C family of RNA-binding proteins and specifically interacts with GLD-2 [16]. Both proteins are located in the cytoplasm of germline and early embryonic cells. In vitro translated recombinant GLD-2 was found to have low levels of PAP activity that could be stimulated by GLD-3 [16,21]. GLD-2 and the other members of the Trf4/5 family lack an RNA-binding domain found in conventional PAPs [21,22]. Thus, GLD-2 and GLD-3 represent a new type of bipartite PAP, where one subunit contributes the catalytic activity and the other the RNA-binding function.

Polyadenylation in eukaryotes has long been considered to be a unique feature of mRNAs. However, there is increasing evidence that polyadenylation also plays a role in the 3′ end processing of noncoding RNA precursors, such as pre-snoRNAs [23] and pre-rRNAs [24]. Furthermore, recent in vivo experiments have implicated Trf4p as a component of an RNA surveillance pathway that involves the polyadenylation of hypomodified initiator tRNA^Met^ (tRNA_i_^Met^) and its subsequent degradation by the nuclear exosome [25]. Mutations in *TRF4,* and in *RRP44,* a gene coding for a subunit of the exosome, were identified as spontaneous suppressors of temperature-sensitive and drug-resistant phenotypes caused by mutations of *TRM6* (*trm6–504). TRM6* encodes a subunit of the enzyme that methylates adenine 58 (m^1^A58) of tRNA_i_^Met^. It was shown that molecules of tRNA_i_^Met^ lacking the methyl group at A58 are prone to degradation and that they could be stabilized by the deletion of Rrp6p [25], a 3′–5′ exonuclease unique to the nuclear form of the exosome (reviewed in [26]). The role of Trf4p in this tRNA surveillance pathway was substantiated by overexpressing Trf4p in *trm6–504* cells, which led to a further reduction of the levels of hypomethylated tRNA_i_^Met^. Because the overexpression of Trf4p in a *trm6–504/*Δ*rrp6* mutant resulted in the accumulation of the polyadenylated form of undermethylated tRNA_i_^Met^, it was proposed that Trf4p was catalyzing this polyadenylation and that the polyadenylation mediated the degradation. However, the authors could not exclude the possibility that Pap1p was responsible for the reaction. The polyadenylation-mediated disposal of aberrant yeast tRNA_i_^Met^ is strikingly similar to the degradation mechanism of aberrant tRNAs and of fragmented mRNAs in E. coli [27–29]. Presumably, poly(A) tails added to any type of RNA in bacteria serve as tags for binding the degradosome (reviewed in [30]).

Here, we report the affinity purification of tagged Trf4p from *S. cerevisiae.* We show that the purified complex has poly(A) polymerase activity provided by the Trf4p subunit and that the activity depends on the zinc knuckle protein Air1p and/or Air2p [31]. Comparison of native tRNA_i_^Met^ with its in vitro transcribed unmodified counterpart demonstrates that the latter RNA is preferentially polyadenylated by the Trf4 complex. The same substrate specificity is maintained by Trf4 complexes reconstituted from recombinantly expressed components. Additional results from experiments with unmodified and native tRNA^Ala^ and a mutant tRNA^Ala^ believed to fold aberrantly, suggest that the Trf4 complex discriminates between correctly and incorrectly folded tRNAs. We also show that the degradation of unmodified tRNA_i_^Met^ by nuclear exosome fractions is dependent on the polyadenylation activity of the Trf4 complex and is stimulated by the putative RNA helicase Mtr4p, which also copurifies with the Trf4 complex. Efficient digestion requires simultaneous polyadenylation by the Trf4 complex, suggesting that the poly(A) tails serve to activate the exosome.

## Results

### Trf4p is a Poly(A) Polymerase

**Figure 1:**
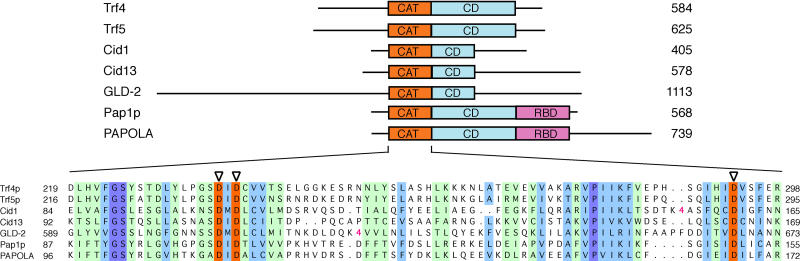
Schematic Alignment of the Domain Organization of Trf4p and Trf5p with Other Members of the Pol-β-like Nucleotidyltransferase Family The proteins are represented as lines, with conserved regions shown as boxes (CAT, catalytic domain; CD, central domain; RBD, RNA-binding domain; sizes in amino acids are indicated to the right). The green boxes in the sequence alignment mark regions with 50% similarity, light blue boxes indicate 100% similarity, and dark blue and orange boxes are regions with 100% identity. The three conserved catalytic aspartates at positions 236, 238, and 294 are in orange and marked by triangles. Regions with four additional amino acids present in Cid1 and GLD-2 are marked with a orange “4.”

Sequence alignments have indicated that Trf4p and Trf5p share several characteristic features with canonical poly(A) polymerases: a catalytic domain at the N-terminus with three conserved aspartate residues, a conserved strand loop motif as found in other template-independent polyribonucleotide polymerases [22,32], and a central domain (Figure 1). In contrast to conventional nuclear poly(A) polymerases and similar to the newly identified cytoplasmic PAPs, Trf4p and Trf5p lack a recognizable RNA-binding domain.

**Figure 2:**
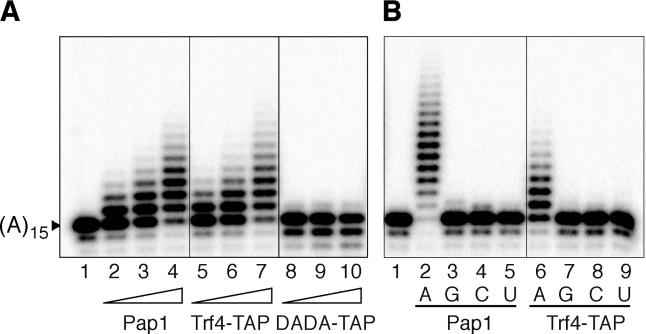
Trf4p Is the Catalytic Subunit of a New Poly(A) Polymerase (A) The Trf4 complex has poly(A) polymerase activity. The 5′-end-labeled oligo(A)_15_ was incubated 30 min with 5, 10, or 20 ng of affinity-purified fractions of the wild-type TAP-tagged Trf4p (Trf4-TAP) or mutant Trf4p with the aspartic acid residues 236 and 238 changed to alanines (DADA-TAP). Protein was omitted in lane 1. Recombinant yeast poly(A) polymerase (Pap1), 1, 2, and 4 ng, was used as a positive control. The migration position of oligo(A)_15_ is indicated by an arrow. (B) The Trf4p activity is specific for the addition of adenosine monophosphate. Polyadenylation assays with 20 ng of Trf4-TAP in the presence of different ribonucleoside triphosphates. Recombinant yeast Pap1p, 5 ng, was used as a control. All samples were separated on 15% denaturing gels.

In order to test whether Trf4p is a bona fide PAP, we affinity-purified Trf4p and assayed the resulting fractions for polyadenylation activity. We used proteinA/[His]_6_-tagged Trf4p expressed from a plasmid in a Δ*TRF4* background strain as well as from a TAP-tagged version (Trf4-TAP; [33]) of the *TRF4* gene inserted into its original chromosomal locus. Affinity-purified Trf4p-containing fractions from either strain were able to extend a synthetic oligo(A)_15_ RNA substrate. Figure 2A shows a polyadenylation assay with Trf4-TAP fractions (lanes 5–7); recombinant yeast Pap1p served as positive control (lanes 2–4). The activity of the Trf4 complex was completely abolished when two of the three highly conserved aspartate residues at positions 236 and 238 in the predicted catalytic domain of Trf4p were mutated to alanines (DADA-TAP; Figure 2A, lanes 8–10). The loss of activity was due to the mutations and not to the instability of the mutant protein because both proteins were expressed at similar levels (data not shown). The reaction catalyzed by Trf4-TAP required ATP, and no activity was detected in the presence of GTP, CTP, or UTP (Figure 2B, lanes 6–9). In a quantitative assay with oligo(A)_15_ as primer ([34]; Materials and Methods), the specific activity of the Trf4 complex was 5.3 × 10^5^ pmol/mg/min. This was similar to the specific activity (5.9 × 10^5^ pmol/mg/min) of recombinant Pap1p measured in parallel. Moreover, incorporation of adenosine monophosphate was strictly dependent on a single-stranded RNA primer (data not shown). These results identified Trf4p as the catalytic component responsible for the polyadenylation activity associated with the purified Trf4 complex. Recombinant Trf4p has been reported to have template-dependent DNA polymerase activity in vitro [18,19]. We therefore tested the Trf4 complex for DNA polymerase activity using different primer–template combinations and Klenow DNA polymerase as control. In contrast to the previous reports, we could not detect any activity (data not shown).

### Trf4p Belongs to an Oligomeric Protein Complex

**Figure 3:**
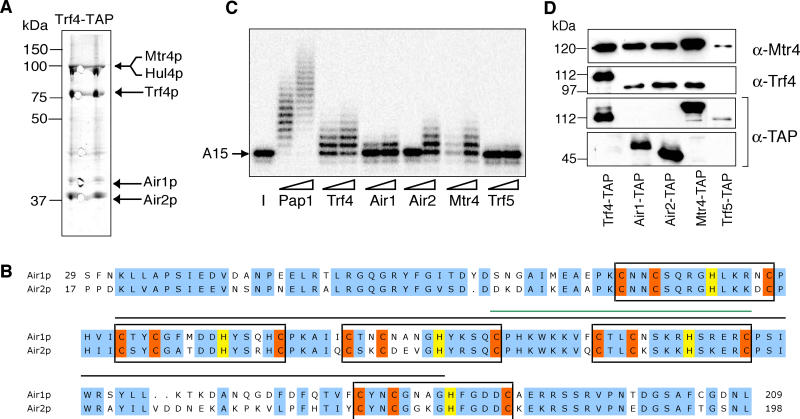
Characterization of the Trf4 Complex (A) Affinity purification of [His]_6_-tagged Trf4 complex identifies five associated polypeptides. Affinity-purified fractions were separated by SDS-PAGE on a 12% gel and stained with Colloidal Coomassie Blue. The protein bands indicated by arrows were identified by MS-MS sequencing. The round holes in the gel lane are a result of punching samples for MS-MS analysis. (B) Alignment of conserved regions of Air1p and Air2p. Boxes indicate five predicted CCHC-type zinc knuckle motifs. Lines above the sequences mark regions of interaction between Air1p (green) or Air2p (black) with Trf4p, as inferred from a yeast two-hybrid screen. (C) Polyadenylation activity associated with TAP-tagged versions of proteins identified as components of the Trf4 complex (Trf4, Mtr4, Air1, and Air2) and of Trf5. The 5′-end-labeled oligo(A_15_) was incubated with 5 or 10 ng of the affinity-purified extracts and analyzed by electrophoresis on a 15% gel. The position of the input RNA is indicated by an arrow. Protein was omitted in lane I. Recombinant yeast poly(A) polymerase (Pap1), 5 or 15 ng, was used as a positive control. (D) Trf4p and Mtr4p copurified with TAP-tagged proteins identified in the Trf4 complex. Western blot analysis of purified eluates with anti-Trf4p and anti-Mtr4p antibody and antibody against the calmodulin-binding region in the TAP-tag (α-TAP). The same amounts of protein complexes as used in the assay (C) were applied to the gel. The higher molecular weight of the Trf4p-TAP protein is due to the TAP-tag. All antibodies were used at a 1:2,000 dilution.

To test whether Trf4p is part of a specific protein complex, affinity-purified Trf4p eluates were analyzed by tandem mass spectrometry (MS-MS). We identified five proteins (Figure 3A): Trf4p; Mtr4p, a putative RNA helicase associated with the nuclear exosome [35]; Hul4p, a putative E3 ubiquitin ligase [36]; and Air1p and Air2p, two zinc knuckle proteins of the CCHC type [31]. As an independent test for the significance of the protein interactions, a yeast two-hybrid analysis was carried out with Trf4p as bait. The screen identified fourteen target sequences: five were fragments of Air1p, four were clones of Air2p, and five were clones of the splicing factor Prp16p ([37]; Table S1). The minimal regions required for the interaction with Trf4p were between amino acid residues 127–149 in Air1p, residues 80–171 in Air2p, and residues 315–503 in Prp16p (Table S1). The minimal Trf4p-interacting regions in Air1p and Air2p overlap (Figure 3B, green and black lines above the alignment), suggesting that both proteins bind to Trf4p in a similar fashion. This region is highly conserved and covers one out of four zinc knuckle motifs in Air1p and three out of four present in Air2p, respectively (Figure 3B).

To confirm the polypeptide composition of the Trf4p-containing complex, we affinity-purified complexes from yeast strains carrying Air1p-TAP, Air2p-TAP, and Mtr4p-TAP. All the “reverse tagged” complexes showed polyadenylation activity (Figure 3C). Western blot analysis confirmed the presence of Trf4p in all complexes, indicating that it is their catalytic moiety (Figure 3D). The Air1-TAP complex contained lower amounts of Trf4p than the other complexes (Figure 3D). This was reflected by a lower polyadenylation activity of Air1-TAP (Figure 3C). Because Trf5p was not found in the Trf4p-TAP complex, we also purified a TAP-tagged version of Trf5p. However, the Trf5p complexes did not show any significant polyadenylation activity (Figure 3C). Instead, we repeatedly observed the incorporation of a single adenosine residue. Compared to the other complexes, Trf5-TAP was expressed less efficiently (Figure 3D). It is not clear whether the weak activity was catalyzed by Trf5p or resulted from traces of contaminating Trf4p or Pap1p undetectable by Western blotting (Figure 3D; results not shown). The role of Hul4p in the Trf4 complex is currently not known.

### Substrate Specificity of the Trf4 Complex

**Figure 4:**
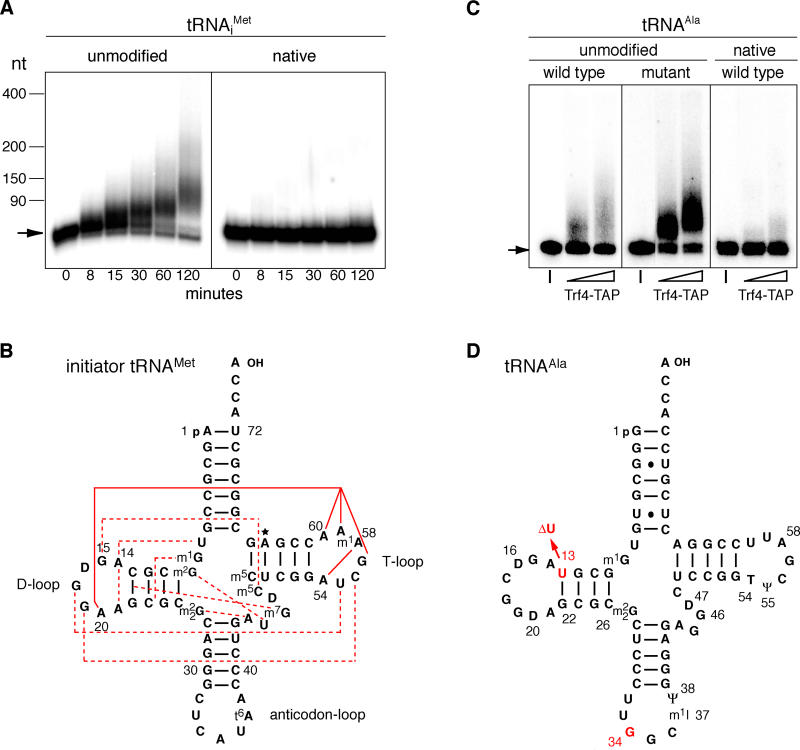
The Trf4 Complex Preferentially Polyadenylates Unmodified tRNA_i_^Met^ and the Unmodified A34GΔU13 Mutant of tRNA^Ala^ (A) Polyadenylation assay with Trf4p-TAP and unmodified and native tRNA_i_^Met^ as substrates. The 5′-end-labeled tRNAs were incubated with 50 ng of Trf4 complex for times indicated and resolved by gel electrophoresis. The migration position of the input tRNA is indicated by an arrow. (B) Sequence of yeast tRNA_i_^Met^ in cloverleaf form with all the known nucleotide modifications. Tertiary interactions are indicated in solid and dashed red lines. The network of hydrogen bonds involving A20, G57, m^1^A58, A59, and A60, necessary and unique for tertiary interactions in eukaryotic initiator tRNA, is outlined by solid red lines. A*, 5′-phosphoribosyl-2′-adenosine; D, dihydrouridine; m^1^A, 1-methyladenosine; m^1^G, 1-methylguanosine; m^2^G, *N^2^*-methylguanosine; m^2^_2_G, *N^2^,N^2^*-dimethylguanosine; m^7^G, 7-methylguanosine; t^6^A, *N^6^*-threonylcarbamoyladenosine. (C) Polyadenylation activity of Trf4p-TAP on unmodified wild-type, A34GΔU13 mutant tRNA^Ala^, and native tRNA^Ala^. The 5′-end-labeled tRNAs were incubated with 20 or 50 ng of Trf4 complex for 30 min at 30 °C. Lane I in each panel shows input tRNA. tRNAs were separated on 10% denaturing gels. (D) Yeast tRNA^Ala^ in its cloverleaf form. All known nucleotide modifications are indicated. In the mutant tRNA^Ala^ used in this study, the uracil at position 13 was deleted, and the A34 in the anticodon loop was changed to guanine (marked in red). ψ, pseudouridine; m^1^I^37^, 1-methylinosine generated by deamination of adenosine at position 37. Other modifications are labeled as in (B).

Prompted by the observation that Trf4p appeared to be responsible for the polyadenylation of hypomodified tRNA_i_^Met^ in vivo, we decided to test unmodified tRNA_i_^Met^ and native tRNA_i_^Met^ as substrates for the Trf4 complex (Figure 4). To this end we used fully modified tRNA_i_^Met^ purified from yeast cells (Figure 4B) and its unmodified counterpart prepared by in vitro transcription. As shown in Figure 4A, the Trf4 complex could selectively polyadenylate the unmodified tRNA, whereas no polyadenylation activity was observed with the native modified tRNA even after long periods of incubation. Within 30 min, poly(A) tails of about 30 nucleotides had been added to unmodified tRNA. The tails continued to grow linearly with time, reaching a length of about 60 to 70 nucleotides after 2 h. To test whether the 3′ hydroxyl end of the native tRNA was accessible, we showed that both tRNA substrates could be polyadenylated with recombinant yeast Pap1p or E. coli PAP (Figure S1).

The methyl group on nucleotide A58 of tRNA_i_^Met^ is required to ensure that the T-loop adopts its correct structure. Thus, the absence of the methyl group at residue A58 of tRNA_i_^Met^ was predicted to interfere with the correct folding of the RNA [25,38]. To test whether the Trf4 complex specifically recognizes any tRNA with impaired tertiary structure, we searched for a tRNA that does not need to be modified in order to fold correctly in vitro and whose conformation could be disrupted by mutation. It has been shown previously that the conversion of adenosine 37 to inosine of in vitro transcribed unmodified yeast tRNA^Ala^ by the editing enzyme Tad1p was abolished by deletion of U13 [39]. This deletion most likely disrupted the last base pair (U13–G22) of the D-stem, and possibly also other crucial tertiary contacts in the neighborhood, such as the triple interaction with G46 (reviewed in [40]; Figure 4D). It was therefore concluded that in vitro transcribed tRNA^Ala^ could adopt an editing-competent tertiary structure in the absence of modifications. We thus employed unmodified wild-type tRNA^Ala^, the ΔU13 mutant, and native tRNA^Ala^ as substrates for the Trf4 complex. Most of the mutated tRNA^Ala^ was polyadenylated, whereas only little activity was observed with the unmodified wild-type tRNA^Ala^ and none with native tRNA^Ala^ (Figure 4C). Taken together, these results suggest that correctly folded tRNAs are poor substrates for the Trf4 complex, whereas aberrantly folded RNAs are efficiently polyadenylated.

### Trf4p Is a Bipartite Poly(A) Polymerase That Can Be Reconstituted from Recombinant Components

**Figure 5:**
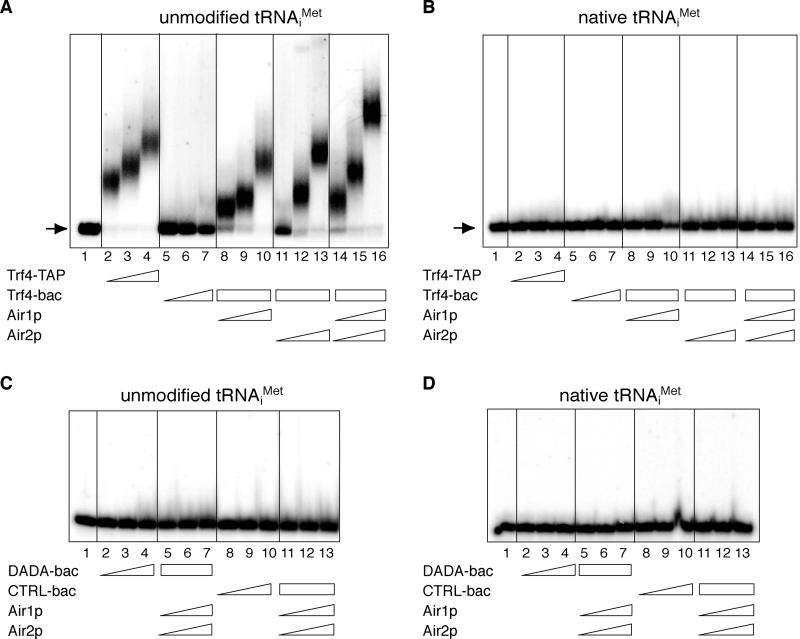
In Vitro Reconstitution of Trf4 Complex from Recombinant Proteins Polyadenylation assays were performed with radiolabeled unmodified (A and C) and native (B and D) tRNA_i_^Met^ substrate incubated with 25, 50, or 100 ng of Trf4-TAP complex (Trf4-TAP; A and B), or with 5, 10, or 20 ng of recombinant Trf4 protein expressed in the baculovirus system (Trf4-bac; A and B), or with 5, 10, or 20 ng of mutant Trf4-bac (DADA-bac; C and D), or with equal amounts and dilutions of control eluates (CTRL-bac; proteins from cell lysates that unspecifically bound to the Ni^2+^-NTA matrix; C and D). In reconstitution experiments 20 ng of Trf4-bac, or DADA-bac, or control eluates were mixed with 0.5, 3, or 15 ng of recombinant Air1p and/or recombinant Air2p in the combinations indicated. The proteins were pre-incubated for 30 min on ice to allow for binding. Reactions were incubated for 50 min at 30 °C. Control reactions contained no protein (lane 1 in each gel).

In order to test which of the proteins identified in the Trf4 complex are required for PAP activity, we aimed to reconstitute the activity from recombinant proteins. Because the recombinant Trf4 protein was insoluble when expressed in *E. coli,* a baculovirus expression system was used to isolate Trf4p from insect cells (Trf4-bac). The affinity-purified Trf4-bac alone showed no poladenylation activity on unmodified tRNA_i_^Met^ (Figure 5A, lanes 5–7). However, polyadenylation was obtained by combining Trf4-bac with either recombinant Air1p or Air2p or both (Figure 5A, lanes 8–16). In these assays, 20 ng of Trf4-bac was mixed with 0.5, 3, or 15 ng of recombinant Air1p (Air1) and/or recombinant Air2p (Air2). The simultaneous addition of both Air1p and Air2p proteins led to the formation of longer poly(A) tails than in reactions containing a single Air protein. This was most likely due to the higher protein concentration in the presence of both proteins. The resulting activities were similar to that of Trf4p-TAP isolated from yeast (Figure 5A, lanes 2–4). Moreover, the activity of the reconstituted complex was specific for unmodified tRNA_i_^Met^ because no significant activity was seen with native tRNA as a substrate (Figure 5B). In a control experiment, the recombinant mutant Trf4p (DADA-bac) containing the same amino acid changes as the double aspartate mutant described above had no polyadenylation activity on either tRNA_i_^Met^, even in the presence of recombinant Air1p and Air2p (Figure 5C and 5D, lanes 2–7). Also, no activity was observed with a control eluate from a Ni^2+^-NTA column to which extracts from uninfected cells had been applied (Figure 5C and 5D, lanes 8–13). These results demonstrated that the Trf4-PAP reconstituted from recombinant proteins retains the ability to selectively polyadenylate unmodified tRNA_i_^Met^.

### Trf4p Stimulates the Activity of the Nuclear Exosome

Next we asked whether the Trf4 complex could directly stimulate the degradation of unmodified tRNA_i_^Met^ by the nuclear exosome in vitro. The fraction of nuclear exosome was prepared by affinity purification of the TAP-tagged Rrp6 protein (Figure S2), an exonuclease exclusively associated with the nuclear and not the cytoplasmic form of the exosome [26]. First, we tested the effect of partially purified Rrp6p-TAP on unmodified tRNA_i_^Met^ (Figure S2C). The Rrp6p-TAP complex showed weak exonuclease activity on this RNA, as indicated by the generation of digestion intermediates and mononucleotide degradation products (Figure S2C, lanes 2–5). This low activity depended on the presence of ATP in the assay (compare lanes 2–5 with 6–9). Most gel lanes showed a labeled band of about 15 nucleotides. We assumed that these molecules resulted from spontaneous cleavage of a small portion of the RNA.

**Figure 6:**
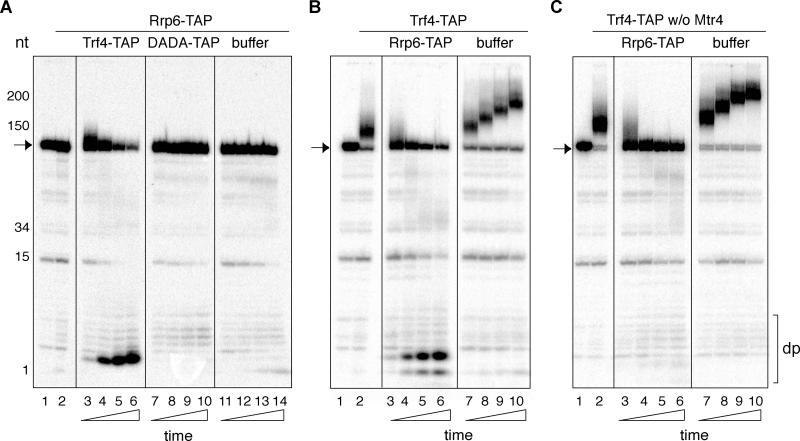
The Polyadenylation Activity of the Trf4 Complex Stimulates the Degradation of Unmodified tRNA_i_^Met^ by the Nuclear Exosome (A) The PAP activity of Trf4 complex is required to stimulate the exosome activity. In a coupled exosome/polyadenylation assay, 5′-end-labeled unmodified tRNA_i_^Met^ was incubated with 50 ng of affinity-purified Rrp6-TAP eluate for 30 min as described in Materials and Methods (lane 2), followed by addition of 50 ng of wild-type (Trf4-TAP), mutant complex (DADA-TAP), or buffer A (buffer). Reactions were stopped after 10 (lanes 3, 7, and 11), 30 (lanes 4, 8, and 12), 60 (lanes 5, 9, and 13), or 90 min (lanes 6, 10, and 14) and separated on a 15% gel. Arrows indicate the position of the input tRNA. Protein was omitted in lane 1 of each gel. The migration positions of the degradation products (dp) are indicated by a bracket. (B) Coupled polyadenylation/exosome assay. The 5′-end-labeled unmodified tRNA_i_^Met^ was pre-adenylated with 50 ng of affinity-purified Trf4-TAP complex for 30 min (lane 2). Then 50 ng of exosome complex (Rrp6-TAP) or buffer A (buffer) was added, and the reactions were continued as in (A). (C) Depletion of Mtr4p results in incomplete degradation. Coupled-assay, 5′-end-labeled unmodified tRNA_i_^Met^ was pre-incubated for 30 min with Trf4p-TAP lacking Mtr4p (Trf4-TAP w/o Mtr4), followed by the addition of 50 ng of Rrp6-TAP complex or buffer A, and the incubation was continued as in (A).

The effect of the Trf4 complex on the activity of the exosome was examined in coupled exosome/polyadenylation assays. Unmodified tRNA_i_^Met^ was pre-incubated with Rrp6-TAP for 30 min. By the end of this time period, a very low amount of degradation products had appeared (Figure 6A, lane 2). Addition of Trf4-TAP resulted in a transient appearance of molecules with short poly(A) tails after 10 min (Figure 6A, lane 3). The extended molecules decreased after this time and had essentially disappeared after 60 min. The decrease of full-length and elongated tRNAs was accompanied by the formation of short degradation products, the majority of which were dinucleotides (Figure 6A, lanes 3–6). Because the RNA substrate was labeled at its 5′ end, the accumulation of dinucleotides indicated that the degradation activity occurred in the 3′ to 5′ direction. This is typical for the nucleases of the exosome [41]. The direction of digestion was further confirmed with 3′ end labeled polyadenylated tRNA_i_^Met^ as substrate. Incubation with Rrp6-TAP resulted in the rapid production of labeled mononucleotides and the concomitant disappearance of the precursors without any detectable intermediates (Figure S3, lanes 2–6). In a control experiment where only buffer was added to Rrp6-TAP fractions, no polyadenylation and no significant degradation of tRNA_i_^Met^ were observed (Figure 6A, lanes 11–14). The possibility that the low amount of degradation observed in the buffer control could be caused by contaminating Trf4-TAP or by copurifying Mtr4p was tested by Western blotting. We could detect no Trf4p and only traces of Mtr4p (Figure S2B). Stimulation of the exosome by the Trf4-TAP complex required the latter's polyadenylation activity because no short degradation products accumulated after the addition of DADA-TAP complexes to Rrp6-TAP eluates (Figure 6A, lanes 7–10). Very similar results were obtained by first polyadenylating tRNA_i_^Met^ with Trf4-TAP and adding Rrp6-TAP in a second step. Almost all the RNA was processed to low molecular weight degradation products within 90 min (Figure 6B, lanes 3–6). The experiment showed that adding longer poly(A) tails to the tRNA prior to starting the coupled reaction did not change the kinetics of the subsequent degradation. In a control experiment, assay buffer was added instead of the exosome fraction. In this case Trf4-TAP continued to elongate the poly(A) tails, which reached an average length of 60–70 nucleotides after 90 min and essentially no degradation was detected (Figure 6B, lanes 7–10). The kinetics of the coupled reactions suggested a processive mode of digestion because only low amounts of partially degraded RNA molecules were generated during the time course.

We then examined the influence of the putative RNA helicase Mtr4p that copurified with the Trf4 complex on the efficiency of digestion by the exosome. We had found previously that the interaction between Trf4p-TAP and Mtr4p could be disrupted by washing the affinity columns with 1 M NaCl prior to elution (Figure S4, lanes 2 and 3). Most likely, the Mtr4p-depleted Trf4-TAP still contained the Air proteins because the fractions retained full PAP activity (Figure 6C, lanes 7–10). In addition, the interaction of the Air proteins with Trf4p was stable upon high-salt treatment of TAP-tagged Air1 or Air2 complexes (data not shown). We used salt-washed eluates of Trf4p lacking Mtr4p to pre-adenylate the tRNA substrate and for subsequent coupled polyadenylation/exosome assays (Figure 6C). Addition of the Rrp6 complexes resulted in the complete removal of the poly(A) tails with time, but the body of the tRNA was not degraded and no significant amounts of small digestion products appeared (compare lanes 3–6 of Figure 6B and 6C). As in the previous experiment, the poly(A) tails continued to grow in the absence of the exosome (Figure 6C, lanes 7–10). These results indicate that Mtr4p is required for the degradation of the tRNA body and support the idea that Mtr4p might function as an RNA helicase to remove the highly ordered tRNA structure.

**Figure 7:**
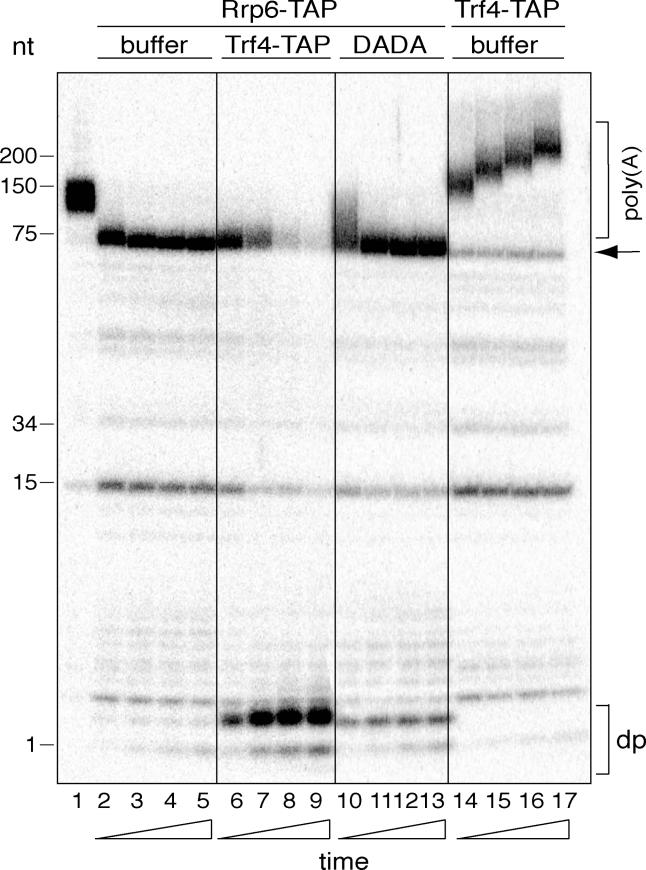
Efficient Degradation of tRNA_i_^Met^ May Involve Multiple Rounds of Deadenylation and Readenylation Uncoupled exosome assay on pre-adenylated tRNA. 5′-end-labeled and in vitro polyadenylated unmodified tRNA_i_^Met^ was incubated with 50 ng of affinity-purified Rrp6-TAP eluate alone or in combination with 50 ng of wild-type (Trf4-TAP) or mutant (DADA) complex or 50 ng of Trf4-TAP alone. Reactions were stopped after 30 (lanes 2, 6, 10, and 14), 60 (lanes 3, 7, 11, and 15), 90 (lanes 4, 8, 12, and 16), or 120 min (lanes 5, 9, 13, and 17) and separated on a 15% gel. The arrow indicates the position of the nonadenylated tRNA. Protein was omitted in lane 1. The migration positions of the polyadenylated tRNA (poly[A]) and the degradation products (dp) are indicated by brackets.

To test whether the presence of a poly(A) tail is sufficient to mediate tRNA degradation we carried out uncoupled exosome assays with pre-adenylated tRNA_i_^Met^. Incubation of such poly(A)-tRNA with Rrp6-TAP resulted in removal of the poly(A) tails only (Figure 7, lanes 2–5). Complete degradation required the combined action of Rrp6-TAP and Trf4-TAP (Figure 7, lanes 6–9). The combination of Rrp6-TAP and mutant Trf4 complex (DADA-TAP) generated only small amounts of dinucleotides (Figure 7, lanes 10–13). This likely reflected a mild stimulation of the exosome by Mtr4p present in DADA-TAP, as detected by Western blotting (data not shown). This experiment showed that the addition of a poly(A) tail is not sufficient for complete degradation of tRNA by Rrp6 complex in the absence of Trf4-PAP activity. As in the previous experiments, Trf4-TAP alone had no nuclease activity above background and elongated the pre-adenylated tRNA during the time course (Figure 7, lanes 14–17).

## Discussion

### Trf4p Is the Catalytic Subunit of a New Heteromeric Yeast Poly(A) Polymerase

Athough a number of new eukaryotic poly(A) polymerases have been discovered in recent years, the functions of most of them are still unknown. Here we describe the properties of Trf4p, the second poly(A) polymerase of S. cerevisiae. Unlike the other newly described PAPs, which are cytoplasmic, Trf4p is located in the nucleus [42]. However, in contrast to the complex multiprotein machinery processing pre-mRNAs associated with canonical yeast Pap1p, Trf4p is part of a small complex in vivo and is capable of polyadenylating tRNAs, products of RNA polymerase III. Lastly, the poly(A) tails made by the Trf4 complex primarily serve to initiate RNA degradation.

The minimal active Trf4-PAP consists of the catalytic subunit Trf4p and either Air1p or Air2p. This minimal complex interacts with Mtr4p, which functionally and physically connects the Trf4-PAP to the nuclear exosome. Although we have identified the Trf4p as the catalytic subunit of the Trf4 complex, the protein is inactive by itself. Polyadenylation activity could be reconstituted by combining recombinant Trf4p and Air1p or Air2p. The Air proteins contain five tandemly arranged zinc knuckles of the CCHC type. Zinc knuckle motifs have been implicated in RNA binding based on mutational and structural studies [43,44]. We therefore suggest that Air1p and Air2p are RNA-binding subunits of the Trf4 complex. This would explain why the addition of recombinant Air1p or Air2p confers activity to the Trf4p subunit, which lacks a recognizable RNA-binding domain. Such a bipartite composition resembles the heterodimeric cytoplasmic PAP of *C. elegans,* in which the GLD-2 protein carries the catalytic activity and GLD-3 is believed to contribute the RNA-binding function [16]. It will be interesting to study whether other noncanonical PAPs are organized in a similar architecture.

The characteristic sequence features of Trf5p and the fact that the *TRF4* and the *TRF5* genes can complement each other and are synthetically lethal when mutated [45] suggest that Trf5p is also a poly(A) polymerase. However, except for an extremely inefficient extension of an oligo(A)_15_ primer, no other RNA substrate has been identified until now. Because Trf5p did not copurify with Trf4-TAP, it is likely that it is assembled into a separate complex.

### The Trf4 Complex May Recognize Structural Features of Misfolded tRNAs

We have shown that the Trf4 complex exclusively polyadenylates unmodified and not native tRNA_i_^Met^. This raised the question of how the Trf4 complex could distinguish between the two otherwise identical RNAs. Residue m^1^A58 has been proposed to be particularly important for specifying a tertiary substructure unique to initiator tRNA [46]. The methylation on a Watson–Crick site forces m^1^A58 to form a symmetric Hoogsteen pair with A54, which promotes the extrusion of A59 and A60 of the loop by stacking with the last base pair of the T-stem (see Figure 4B). This conformation of the T-loop induces the specific interactions between the D- and T-loops that are necessary to stabilize the tRNA's tertiary structure [46]. This may explain why tRNA_i_^Met^ is particularly sensitive to the absence of the methyl group on residue A58 compared to other tRNAs containing m^1^A58 [25,46]. The lack of this methyl group presumably results in a change of the overall shape of the tRNA and a less compact conformation of the T- and D-hairpins. Exposed stem-loops of the hypomethylated tRNA could serve as recognition elements for the Trf4 complex. This model predicts that the Trf4 complex monitors structural features and not the modification state of the tRNAs. Aberrant folding of the tRNA is recognized and leads to the selective activation of the polyadenylation reaction. This idea is further supported by our results obtained with alanine tRNAs. These tRNAs are thought to fold correctly without the need for modifications [39]. Accordingly, both unmodified and native wild-type tRNA^Ala^ were poor substrates for the Trf4 complex. In contrast, the ΔU13 mutant was polyadenylated very efficiently, presumably because of its altered tertiary structure.

While finding this model attractive, we realize that experimental proof will require much further work. Probing the structural features of the different forms of tRNAs by chemical and enzymatic methods may help to elucidate the mechanism of RNA recognition. Ultimate proof should be obtained by determining the structure of RNA–protein complexes. The reconstituted recombinant Trf4 complexes showed the same preference for unmodified tRNA substrates as found with Trf4p-TAP from yeast. It is surprising that the substrate preference for unmodified tRNA_i_^Met^ could be provided either by Air1p or by Air2p. We assume that Trf4p can form two different complexes containing Air1p or Air2p as alternative subunits. The prediction that the Air proteins are the RNA-binding subunits of Trf4-PAP implies that they not only contribute the RNA-binding functions but also the differential substrate recognition. Future analysis of the RNA-binding properties and the kinetic parameters of the Trf4-catalyzed reaction should help to confirm this prediction and elucidate the mechanism of substrate recognition.

### The Trf4 Complex Stimulates the Nuclear Exosome In Vitro

We have shown that the polyadenylation activity of the Trf4 complex stimulates the activity of the exosome on unmodified tRNA_i_^Met^ in vitro. In agreement with previous work [41], the nuclear exosome complex purified from yeast had very low activity on its own. It has been proposed that the activation of the exosome requires additional factors (reviewed in [26]). Because Trf4 complexes contain Mtr4p, it is reasonable to assume that this protein mediates the stimulation by attracting the exosome to the tRNA-bound Trf4 complex. As shown here, Mtr4p is needed for the exosome to digest through the structured regions of the tRNA substrates. However, Mtr4p alone (data not shown) or provided by the complex of mutant DADA-TAP was not sufficient to promote tRNA degradation*.* Instead, we demonstrated that the activation of the exosome completely depended on both the polyadenylation activity of the Trf4 complex as well as on the presence of Mtr4p. Moreover, the last experiment implied that complete degradation of the RNA needs the cooperative activity of both the Trf4 and the Rrp6 complexes. This interplay may involve continuous cycles of poly(A) addition and removal before the exosome moves into the structured parts of the tRNA substrates.

The mechanism of the degradation of unmodified yeast tRNA in vitro described above and the model proposed by Kadaba and coworkers [25] is similar to the quality control mechanism disposing of aberrant tRNAs and the pathways of mRNA turnover in E. coli [28,30,47]. In eubacteria, tRNA surveillance also depends on polyadenylation and on subsequent degradation by 3′–5′ exonucleases, components of the degradosome. Furthermore, many of the eukaryotic exosome components are related to the enzymes of the bacterial degradosome (reviewed in [26,48,49]). We speculate that the prokaryotic paradigm of PAP-mediated RNA turnover represents the ancient role of polyadenylation and that the stabilization function of poly(A) tails evolved as a consequence of the compartmentalization during the evolution of eukaryotic cells. The fact that Trf4-PAP-mediated polyadenylation resembles the system operating in prokaryotes and that homologs of Trf4p can be identified in all eukaryotic lineages (S. V., unpublished data) implies that the two polyadenylation pathways operate simultaneously in extant eukaryotes.

Moreover, it has been shown that under certain conditions exosome-processed precursors to many mRNAs and stable RNAs accumulate as polyadenylated molecules [23,24,50]. It has been suggested that this polyadenylation requires the canonical nuclear Pap1p; even so, the reactions occur independent of the pre-mRNA cleavage and polyadenylation machinery. It will be interesting to examine whether the Trf4 complex is involved in the polyadenylation-mediated processing of some of these RNAs or whether its substrate repertoire is restricted to aberrant tRNA molecules.

## Materials and Methods

### 

#### Plasmids

The following plasmids were used in this study: WK337 (TRF4-pBluescript KS+), WK339 (wt TRF4-pNOPPATA), WK340 (DADA-TRF4-pNOPPATA), AIR1-pET22a(+), and AIR2-pET22a(+). PAP1p was expressed from plasmid pGM10-YPAP-tag1 and E. coli PAP from a plasmid obtained from Dr. Mario Mörl, Max-Planck-Institute for Evolutionary Anthropology, Leipzig, Germany.

#### Construction of plasmids

Yeast genomic DNA was used to PCR-amplify the coding regions of *TRF4, AIR1, AIR2, MTR4,* and *HUL4.* The full-length ORF of *TRF4* (YOL115W) was amplified by PCR with primers encoding 6× His in frame with the C-terminus of Trf4p. The sequence of the primers used can be obtained upon request. The PCR product was subcloned into the NdeI and SalI restriction sites of the p-Bluescript II KS+ plasmid (Stratagene, La Jolla, California, United States). The TRF4-DADA mutant was generated by site-directed mutagenesis of wild-type *TRF4* in p-Bluescript II KS+. Correct cloning and mutagenesis was confirmed by sequencing. Both inserts were subsequently subcloned to a yeast expression vector (pNoppata) encoding an N-terminal protein A tag and a TEV cleavage site and digested with NdeI and SalI restriction enzymes, resulting in the plasmids WK339 (wild-type *TRF4*) and WK340 (mutant *TRF4*).

The recombinant Air1 and Air2 proteins for the in vitro reconstitution of the Trf4 complex were inserted into the NdeI and XhoI sites of the pET22a(+) expression vector (Novagen, Madison, Wisconsin, United States), expressed in BL21(DE3) and purified on Ni^2+^-NTA columns. All constructs were verified by sequencing.

#### Plasmids for yeast two-hybrid analysis.

The full-length coding region (ORF) of Trf4p (YOL115W) was amplified from genomic DNA of S. cerevisiae with the primers Trf4.13 (5′-aaaaccgcggccatgggggcaaagagtgtaaca-3′) and Trf4.14 (5′-aaattaattaaattaaagggtataaggattatatcc-3′). The PCR products were digested with PacI, filled in with Klenow DNA polymerase and digested with SacII. The purified DNA fragments were inserted into the corresponding restriction sites on the pB27 plasmid (Hybrigenics, Paris, France). Correct cloning was confirmed by sequencing.

#### Yeast strains

Manipulations and growth of Saccharomyces cerevisiae were performed by standard procedures. Genotypes of strains used are as follows: JW72 (*Mat a; BY4741; his3*Δ*1; leu2*Δ*0; met15D0; ura3*Δ*0; YOL115w::kanMX4;* [pWK336; *TRF4*-pNoppata]); and JW73 (*Mata; BY4741 his3*Δ*1; leu2*Δ*0; met15*Δ*0; ura3*Δ*0; YOL115w::kanMX4;* [pWK340; Trf4-DADA-pNOPPATA]). Strains with C-terminal TAP-fusion of *TRF4* (YOL115W), *AIR1* (YIL079C), *AIR2* (YDL175C), *TRF5* (YNL299W), *MTR4* (YJLO5OW), *RRP6* (YOR001W), and *PAP1* (YKR002W) isogenic to S288C ( ATCC 201388:*MATa his3*Δ*1 leu2*Δ*0 met15*Δ*0 ura3*Δ*0;* Open Biosystems, Huntsville, Alabama, United States) were purchased from BioCat (Heidelberg, Germany). The correct integrations of the TAP-tag were verified by PCR according to the manufacturer's instructions. To construct JW72 and JW73, plasmids pWK339 and pWK340 were transformed into the *trf4*Δ strain (clone ID 6265, isogenic to BY4741, Research Genetics, Invitrogen, Carlsbad, California, United States).

#### Yeast two-hybrid analysis

The Trf4p clone used for the yeast two-hybrid screen is described above. The two-hybrid tests were carried out under the auspices of the EEC (grant RNOMICS, QLG2-CT-2001–01554) by Hybrigenics as described [51].

#### Affinity purification of protein complexes

Yeast extracts were prepared from 2-l cultures grown to an OD_600_ of 2.0–3.0 as described [52]. The purifications on IgG Sepharose were done as described [53]. Samples were eluted and stored in buffer A (50 mM Tris (pH 7.9), 120 mM KCl, 0.02% NP-40, 0.5 mM EDTA, 1 mM DTT, 10% glycerol, and protease inhibitors [0.5 μg/ml Leupeptin, 0.8 μg/ml Pepstatin A, and 0.6 mM PMSF]). For further purification on Ni^2+^-NTA agarose, peak fractions were pooled and mixed 1:1 with buffer B (50 mM Tris (pH 7.9), 10% glycerol, 150 mM KCl, 0.02% NP-40, 2 mM β-mercaptoethanol, protease inhibitors as in buffer A) and bound to 1/10 of total volume of Ni^2+^-NTA agarose. Samples were washed five times by agitation with one volume of binding buffer containing 10 mM imidazole. Proteins were released with elution buffer containing 250 mM imidazole and dialyzed against buffer B containing 1 mM dithiothreitol instead of β-mercaptoethanol.

#### Identification of polypeptides in the Trf4 complex

Matrix-assisted laser desorption ionization time of flight (MALDI-TOF) mass spectrometry was conducted. Colloidal-Coomassie-Blue-stained bands of interest were in-gel digested with trypsin as described [54]. After overnight digestion, about 1 μl was mixed with 1 μl of saturated alpha-cyano cinnamic acid in 50% acetonitrile/0.1% trifluoroacetic acid in water and applied to the MALDI-target. The samples were analyzed with a Bruker Daltonics (Bremen, Germany) Ultraflex TOF/TOF mass spectrometer. An acceleration voltage of 25 kV was used. Calibration was internal to the samples with des-Arg-bradykinin and ACTH(18–38) (both peptides purchased from Sigma, St. Louis, Missouri, United States).

#### Nanoelectrospray ionization MS-MS.

For this approach the peptides obtained after tryptic digestion were desalted and further concentrated on a pulled capillary containing approximately 100 nl of POROS R2 reverse phase material (Applied Biosystems, Framingham, Massachusetts, United States). The peptides were eluted with about 1 μl of 60% acetonitrile in 5% formic acid directly into the nanoelectrospray capillary needle. Mass spectra were acquired on a QSTAR Pulsar I quadrupole TOF tandem mass spectrometer (Applied Biosystems/MDS-Sciex, Toronto, Canada) equipped with a nanoelectrospray ion source (Proxeon, Odense, Denmark) as described [55]. Fragmentation by MS-MS yields a stretch of amino acid sequence together with its location in the peptide (sequence tag). With this sequence tag information appropriate databases (e.g., MASCOT) were searched.

#### Recombinant protein expression and purification

E. coli BL21 or M15 cells transformed with the respective plasmids were grown in 2xYT medium at 25 °C to an OD_600_ of 1.0. After induction with 0.4 mM IPTG, incubation was continued for 10 h. Proteins carrying a C-terminal [His]_6_ tag were purified on Ni^2+^-NTA agarose (Sigma) according to the manufacturer's instructions.

#### Expression of proteins with the baculovirus system

The open reading frame of Trf4p was cloned into the StuI and XhoI sites of the pFASTBAC-vector HTb that contains a [His]_6_ affinity tag at both ends. Proteins were expressed with the BAC-To-BAC expression system (Invitrogen) according to the manufacturer's protocol. A total of 1 × 10^6^ Sf9 cells were seeded per 35-mm well of a six-well plate in 2 ml of Sf-900 II SFM (Gibco, San Diego, California, United States) containing penicillin/streptomycin and transfected with approximately 5 μl of bacmid DNA. After 48 h at 27 °C, the virus was used to infect 2 × 10^6^ cells that had been seeded on a 10-ml plate. After 4–5 d, 2 ml of the newly produced virus was added to a 30-ml suspension culture with 1 × 10^6^ Sf9 cells/ml. The virus was collected 3–5 d later and used to infect 100-ml cultures. After 4–5 d the cells were harvested and washed with 1× PBS. The pellet was resuspended in approximately two packed volumes of lysis buffer (200 mM NaCl, 50 mM Tris, 10% glycerol 0.02 % NP40, 0.5 mM PMSF, 0.7 μg/ml Pepstatin, and 0.4 μg/ml Leupeptin) and incubated on ice while shaking for 30 min. The lysate was centrifuged for 30 min at 10,000 rpm at 4 °C. The supernatant containing recombinant proteins carrying a [His]_6_ affinity tag was then incubated with an appropriate amount of Ni^2+^-NTA resin on a rotor arm in the cold room for approximately 1 h. Proteins attached to the Ni^2+^-NTA beads were either batch-purified or applied to a column and washed with lysis buffer. Elution was done with lysis buffer containing 250 mM imidazole, and proteins were analyzed by SDS-PAGE followed by Western blot analysis.

Protein concentrations were determined with a commercial protein assay kit (Bio-Rad, Hercules, California, United States), and protein amounts in SDS-PAGE were estimated by comparison to BSA standards.

#### Antibodies

A C-terminal portion of Trf4p (aa 356–584) was cloned into the pQE-9 vector and expressed in the E. coli strain M15pREP4 (Qiagen, Valencia, California, United States). The resulting C-terminal Trf4p fragment contained a [His]_6_ tag fusion on its C-terminus. The protein was expressed according to the manufacturer (Qiagen) and affinity-purified on Ni^2+^-NTA agarose (Sigma) under denaturing conditions as described. Approximately 100 μg of purified protein was used for three injections into a rabbit (Eurogentec, Seraing, Belgium). Mtr4 antibodies were kindly provided by Dr. Patrick Linder (University of Geneva). The Anti-TAP antibody against the C-terminal region of the TAP-tag were from Open Biosystems.

#### Purification of native tRNAs

About 20% pure fractions of tRNA_i_^Met^ and 80% enriched fractions of tRNA^Ala^ were prepared from S. cerevisiae by counter-current distribution [56]. tRNA_i_^Met^ was further purified to near homogeneity by column chromatography on Sepharose 4B and BD-cellulose under conditions similar to those described previously [57], followed by 5′ end labeling and gel purification. tRNA^Ala^ was gel purified after 5′ end labeling.

#### In vitro transcription and RNA labeling

A plasmid ptRNA^Met^ for the in vitro transcription of yeast tRNA_i_^Met^ [58] was obtained from Dr. Bruno Senger (IBMC Strasbourg). The plasmid was linearized with BstNI and transcribed with T7 RNA polymerase. The plasmid for the transcription of sCYC1 [59] was linearized with EcoRI and transcribed with SP6 RNA polymerase. The RNAs for mutant and wild-type tRNA^Ala^ were in vitro transcribed as described [39]. All transcripts were purified on 8.3 M urea/12% polyacrylamide gels. For 5′ end labeling, RNAs were treated with alkaline phosphatase and labeled with γ-^32^P-ATP (Amersham Pharmacia, Piscataway, New Jersey, United States) and T4 polynucleotide kinase. Labeled RNAs were purified as described [60].

#### Renaturation of tRNAs.

RNA pellets were dissolved in RNase-free water, incubated for 1 min at 65 °C, followed by incubation for 10 min on ice after the addition of an equal volume of twice-concentrated assay buffer containing 10 mM MgCl_2_ (see below).

#### Polyadenylation assays and reactions

Polyadenylation assays were carried out in 10- to 25-μl reaction mixtures containing 5–50 ng of affinity-purified protein, 50 fmol of 5′-end-labeled RNA, 0.5 mM ATP, 5 mM MgCl_2_, 25 mM Tris-HCl (pH 7.9), 20 mM KCl, 10% glycerol, 0.01 mM EDTA, 0.1 mg/ml BSA, 1 mM DTT, 0.02% Nonidet P-40, and 5U of RNA guard (Promega, Fitchburg, Wisconsin, United States). Reactions were incubated at 30 °C for the times indicated and stopped by the addition of 25 mM EDTA. The RNA was precipitated by adding 0.1 volumes of 3M ammonium acetate and three volumes of ethanol. Pellets were resuspended in 6 μl of formamide loading buffer and separated on denaturing polyacrylamide gels. Radioactivity was scanned with a PhosphorImager and results analyzed with ImageQuant software (Molecular Dynamics, Sunnyvale, California, United States).

The specific activities of poly(A) polymerases were measured in 10-μl reaction mixtures containing 20 mM Tris-Cl (pH 8.4), 40 mM KCl, 5 mM MgCl_2_, 2 mM DTT, 0.01% NP-40, and 0.1 mg/ml BSA with 0.5 mM ATP, 0.1 μCi [α-^33^P]ATP (3,000 Ci/mmol), and 5 μM (A)_15_ or unmodified tRNA_i_^Met^.Recombinant yeast Pap1p [34] or Trf4p-TAP complex, 2, 4, or 8 ng, containing 1, 2, or 4 ng of Trf4 protein was incubated for 20 min at 30 °C. The reactions were stopped by spotting onto a 1.5-cm^2^ DE-81 paper. The filters were washed 3× 10 min in 0.3 M ammonium formate/10 mM Na-pyrophosphate, and the incorporated radioactivity was measured in a scintillation counter.

Polyadenylation of tRNA for uncoupled exosome assays was done in 50-μl reaction volumes containing 1 μg of recombinant Pap1p in the presence 100 ng of 5′-end-labeled tRNA, 50 μM ATP, 5 mM MgCl_2_, 25 mM Tris-HCl (pH 7.9), 20 mM KCl, 10% glycerol, 0.01 mM EDTA, 0.1 mg/ml BSA, 1 mM DTT, 0.02% Nonidet P-40, 5U of RNA guard (Promega), and 100 ng of heparin. Reactions were incubated at 30 °C for 30 min and stopped by the addition of 25 mM EDTA. The RNA was precipitated by adding 0.1 volumes of 3 M ammonium acetate and three volumes of ethanol. Pellets were resuspended in 6 μl of formamide loading buffer and separated on denaturing polyacrylamide gels. The polyadenylated population of tRNAs was purified as described [60].

#### Exosome assays

Exosome assays were carried out under the same conditions used for polyadenylation assays except that the reactions contained 1 mM ATP and 2 mM DTT. To prevent loss of small degradation products during RNA precipitation, the reactions were stopped by the addition of an equal volume of gel loading buffer containing 80% formamide and separated by electrophoresis without prior precipitation.

#### Database searches and sequence analysis

BLAST searches were conducted in the genome databases (http://www.ncbi.nlm.nih.gov/sutils/genom_table.cgi) or other nonredundant or EST databases at the Swiss EMBnet node [61] (http://www.ch.embnet.org/software/BottomBLASTadvanced.html) or at the NCBI (http://www.ncbi.nlm.nih.gov/BLAST/).

## Supporting Information

Figure S1 Native tRNA_i_^Met^ Can Be Polyadenylated by the Activity of Recombinant Yeast Pap1p and Eubacterial PAP Polyadenylation activity of Pap1p, Trf4p-TAP, and E. coli PAP (Eco-PAP) on native and unmodified tRNA_i_^Met^. The 5′-end-labeled tRNAs were incubated with 50 ng of Trf4 complex, 50 ng of Pap1p, or 50 ng of E. coli PAP for the times indicated and separated on a 10% gel. The migration position of the input tRNA is indicated by an arrow. Lane I on each gel contained input tRNA alone.
(871 KB PDF).

Figure S2 Composition and Activity of the Rrp6-TAP Complex (A) Polypeptide composition of the affinity-purified TAP-tagged Rrp6p (Rrp6-TAP). The purification was done as described in Materials and Methods, and a sample was separated by 12% SDS-PAGE and stained with silver. The predicted position of Rrp6p is indicated by an arrow.
(B) Rrp6p-TAP eluates contain residual amounts of Mtr4p. Western blot analysis of Rrp6-TAP with antibodies directed against Mtr4p, Trf4p, and the C-terminus of the TAP-tag. Affinity-purified fractions of Mtr4p (Mtr4-TAP) were used as a control.
(C) The Rrp6p-TAP complex alone does not efficiently degrade unmodified tRNA_i_^Met^. The 5′-end-labeled in vitro transcribed tRNA_i_^Met^ was incubated with 100 ng of Rrp6p-TAP complex in the presence of 1 mM ATP (lanes 2–5) or without ATP (lanes 6–9). Reactions were stopped after 10, 30, 60, or 120 min by the addition of one volume of formamide loading buffer and analyzed by electrophoresis on a 15% gel. The position of the input RNA is indicated by an arrow.
(1.2 MB PDF).

Figure S3 The Rrp6p-TAP Complex Has 3′ to 5′ Exonuclease Activity In vitro transcribed tRNA_i_^Met^ was polyadenylated with recombinant yeast Pap1p; the polyadenylated products were eluted from polyacrylamide gel and labeled at their 3′ ends with α-^32^P cordycepin triphosphate (3′ dATP, NEN). The poly(A)-tRNA_i_^Met^ was then incubated with 100 ng of Rrp6p-TAP complex (lanes 2–6) or with buffer A (lanes 7–11) for 2, 15, 30, 45, or 60 min, stopped by the addition of one volume of formamide loading buffer, and analyzed by electrophoresis on a 15% gel.
(750 KB PDF).

Figure S4 Mtr4p Can Be Removed from TAP-Tagged Trf4 Complexes by High Salt Western blot analysis of 100 ng of Trf4-TAP and Mtr4-TAP complexes purified on IgG Sepharose at low salt (L) and high salt (H) conditions. Lane W on each gel shows samples eluted by 1 M NaCl from the affinity columns prior to TEV protease treatment.
(337 KB PDF).

Table S1 Proteins Interacting with Full-Length Trf4p in a Yeast Two-Hybrid Screen “Start” and “stop” indicate regions of proteins expressed in particular clones. Numbers in brackets indicate that the clone extended beyond the open reading frame. Global predicted biological score (PBS) is a confidence score assigned to each interaction by Hybrigenics, which predicts the occurrence of an interaction in vivo [62].
(27 KB DOC).

### Accession Numbers

The Structural Genomics Consortium (http://www.sgc.ox.ac.uk/) accession numbers for genes and gene products discussed in this paper are Air1p (SGDID:S000001341), Air2p (SGDID:S000002334), Hul4p (SGDID:S000003797), Mtr4p (SGDID:S000003586), Pap1p (SGDID:S000001710), Prp16p (SGDID:S000001794), *RRP44* (SGDID:S000005381), Rrp6p (SGDID:S000005527), Tad1p (SGDID:S000003212), Trf4p (SGDID:S000005475), Trf5p (SGDID:S000005243), and *TRM6* (SGDID:S000005006). The Swiss-Prot/EMBL (http://www.ebi.ac.uk/swissprot/) accession numbers for genes and gene products discussed in this paper are Cid1 (*S. pombe;* O13833), Cid13 (*S. pombe;* Q9UT49), GLD-2 (isoform a, *C. elegans;* O17087), GLD-3 (*C. elegans;* Q95ZK6), PAP (*E. coli;* P13685), Pap1p (*S. cerevisiae;* P29468), PAPOLA (bovine; P25500), Trf4 (*S. cerevisiae;* P53632), and Trf5 (*S. cerevisiae;* P48561).

## Abbreviations

ATP: adenosine triphosphate
MS-MS: tandem mass spectrometry
PAP: poly(A) polymerase
pre-mRNA: messenger RNA precursor
TAP: tandem affinity purification
tRNA_i_^Met^: initiator tRNA^Met^
