# Supplementary material for: A New Yeast Poly(A) Polymerase Complex Involved in RNA Quality Control
Source: PLoS Biol. 2005 Apr 19;3(6):e189. doi: 10.1371/journal.pbio.0030189 (PMC1079787; doi:10.1371/journal.pbio.0030189)
Supplement: Supplementary file 1 [file pbio.0030189.sg001.pdf]

Figure S1

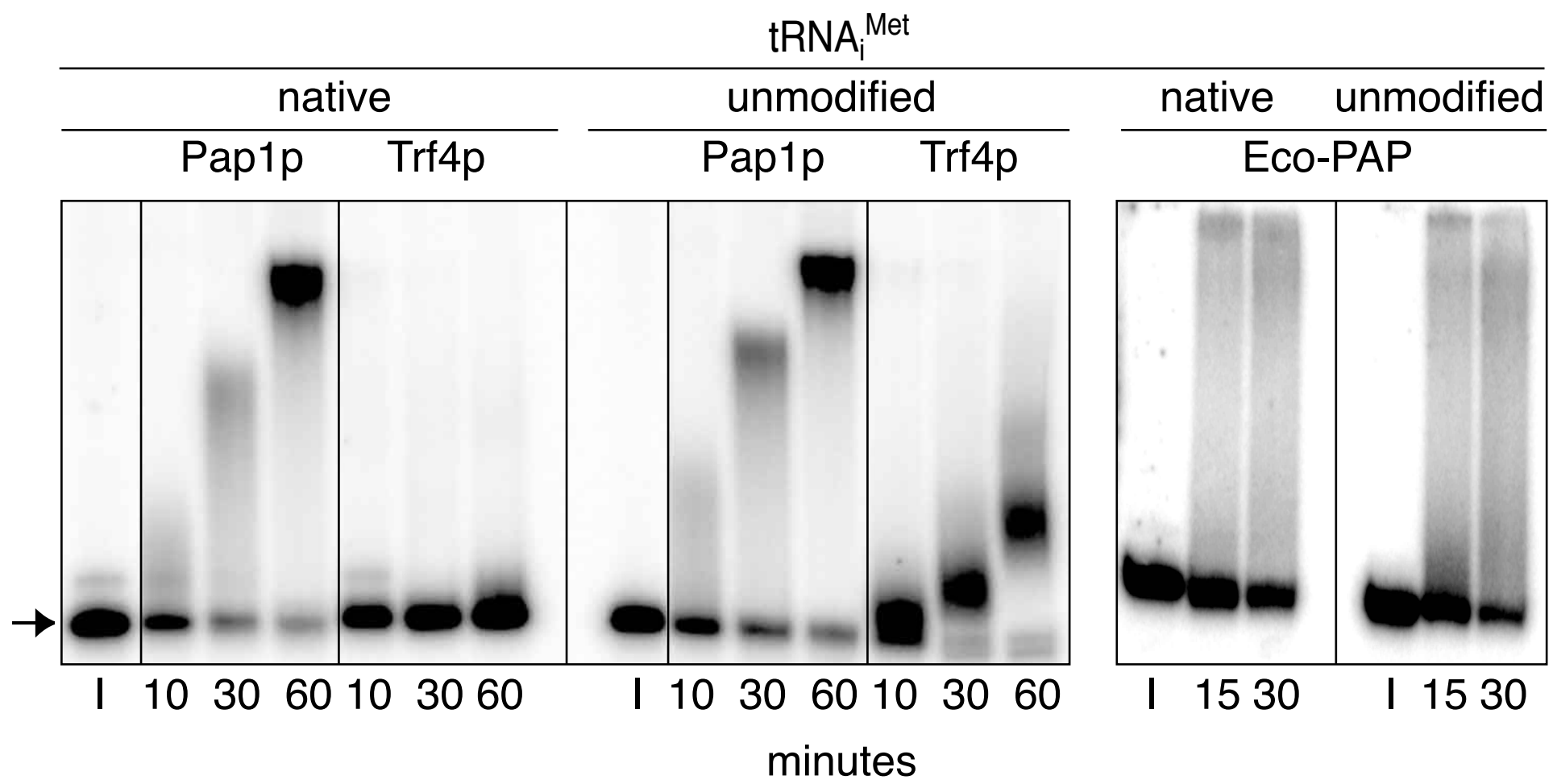

tRNAiMet
native
unmodified
Pap1p
Trf4p
Pap1p
Trf4p
native
unmodified
Eco-PAP
I 10 30 60 10 30 60
I 10 30 60 10 30 60
I 15 30
I 15 30
minutes
