# Supplementary material for: A New Yeast Poly(A) Polymerase Complex Involved in RNA Quality Control
Source: PLoS Biol. 2005 Apr 19;3(6):e189. doi: 10.1371/journal.pbio.0030189 (PMC1079787; doi:10.1371/journal.pbio.0030189)
Supplement: Supplementary file 2 [file pbio.0030189.sg002.pdf]

Figure S2

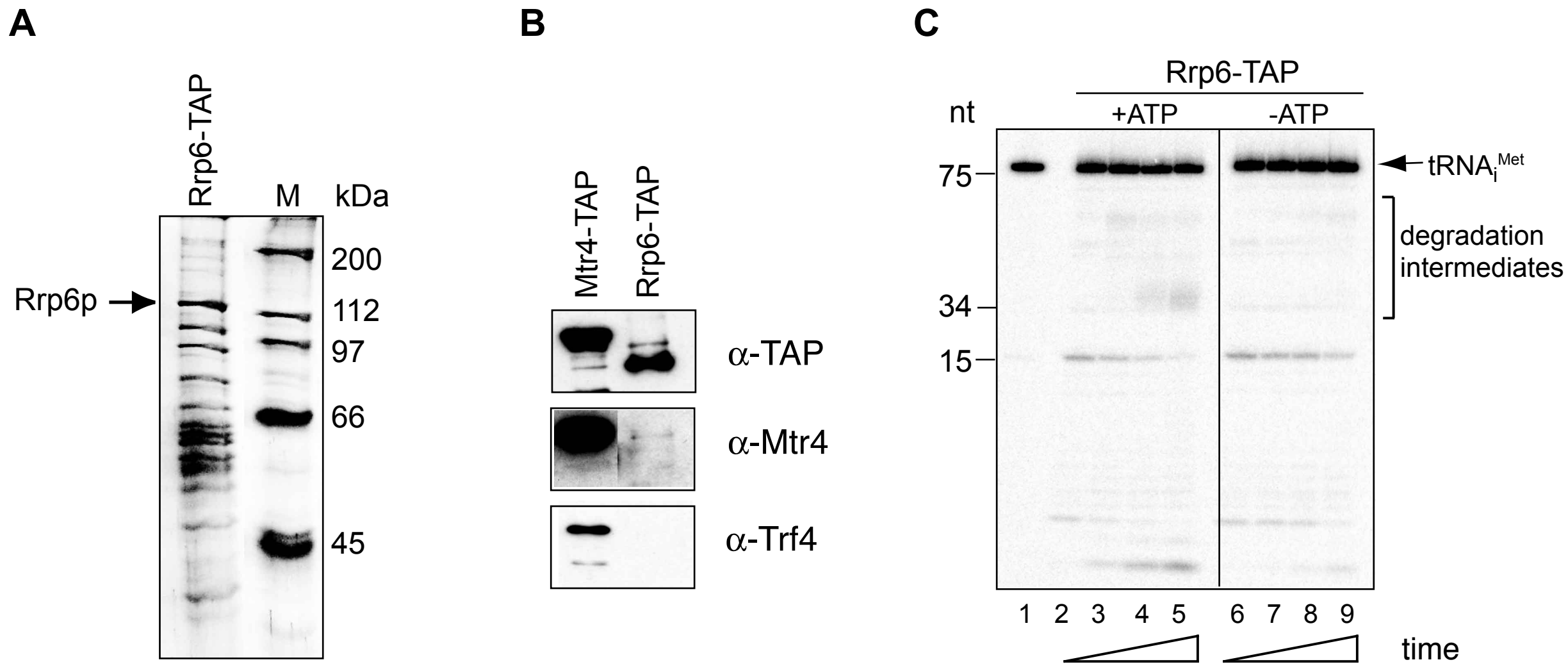
A
Rrp6-TAP
M
kDa
Rrp6p
200
112
97
66
45
B
Mtr4-TAP
Rrp6-TAP
α-TAP
α-Mtr4
α-Trf4
C
Rrp6-TAP
nt
+ATP
-ATP
75
34
15
tRNAi Met
degradation intermediates
1 2 3 4 5 6 7 8 9
time
