# Supplementary material for: A New Yeast Poly(A) Polymerase Complex Involved in RNA Quality Control
Source: PLoS Biol. 2005 Apr 19;3(6):e189. doi: 10.1371/journal.pbio.0030189 (PMC1079787; doi:10.1371/journal.pbio.0030189)
Supplement: Supplementary file 5 [file pbio.0030189.st001.doc]

Table S1

Proteins interacting with Trf4p in yeast-two hybrid analysis.
